# Anti-inflammatory effects of 3PO in asthmatic airway inflammation: an integrated study using network pharmacology, molecular modelling, and *in vivo* experiments

**DOI:** 10.3389/fphar.2026.1790273

**Published:** 2026-04-02

**Authors:** Siqing Huang, Shuna Wei, Fang Luo, Yuanxiong Cheng

**Affiliations:** 1 The Second School of Clinical Medicine, Southern Medical University, Guangzhou, China; 2 Department of Pulmonary and Critical Care Medicine, The Affiliated Guangdong Second Provincial General Hospital of Jinan University, Guangzhou, China; 3 The First Clinical Medical College, Lanzhou University, Lanzhou, China; 4 Department of Pulmonary and Critical Care Medicine, Gansu Provincial Hospital, Lanzhou, China; 5 Department of Pulmonary and Critical Care Medicine, The Second School of Clinical Medicine, Southern Medical University, Guangzhou, China

**Keywords:** 3PO, airway inflammation, asthma, NF-κB signaling pathway, PFKFB3

## Abstract

**Introduction:**

Recent studies have highlighted the critical role of 6-phosphofructo-2-kinase/fructose-2,6-bisphosphatase-3 (PFKFB3) in inflammation, with inhibitors like 3PO showing therapeutic potential in various inflammatory diseases. However, its effect on asthma inflammation remains unclear.

**Methods:**

Our study employed an integrative strategy—combining network pharmacology, molecular docking, molecular dynamics simulations, and *in vivo* experiments—to investigate 3PO’s anti-inflammatory action in asthma.

**Results:**

Firstly, the construction of a protein-protein interaction (PPI) network identified nine hub genes: TNF, IL6, BCL2, IL1B, CASP3, RELA, MAPT, MAOB, and MAOA. Then, the KEGG pathway enrichment analysis identified the nuclear factor kappa B (NF-κB) pathway as a pivotal target. Molecular docking and dynamics simulations suggested stable interactions between 3PO and key inflammatory markers, including tumor necrosis factor-α (TNF-α), interleukin-1β (IL-1β), interleukin-6 (IL-6), and NF-κB P65. For experimental validation, the OVA-induced asthma mouse models were established. PFKFB3 expression was markedly upregulated in the lung tissues of asthmatic mice. 3PO treatment modulated mediator levels in BALF of asthmatic mice by reducing interleukin-4 (IL-4), interleukin-5 (IL-5), interleukin-13 (IL-13), interleukin-17A (IL-17A), IL-1β, TNF-α, IL-6, lactate, Immunoglobulin E (IgE), and chemokine (C-C motif) ligand 11 (CCL11), while elevating interleukin-10 (IL-10). 3PO treatment alleviated hallmark features of asthma in this model, such as airway mucus hypersecretion, goblet cell hyperplasia, and peribronchial infiltration of inflammatory cells, particularly eosinophils. Furthermore, 3PO treatment reversed key protein changes in lung tissue by increasing inhibitor of nuclear factor Kappa B-α(IKB-α) and decreasing p-NF-κB P65.

**Discussion:**

These results indicate that 3PO exerts anti-inflammatory effects in asthma, and this process is associated with the suppression of the NF-κB pathway, providing a novel therapeutic rationale for asthma therapy.

## 1 Introduction

Bronchial asthma is a chronic respiratory disease characterized by recurrent wheezing, chest tightness, cough, and shortness of breath with varying degrees of expiratory airflow limitation (GINA, 2025). Its main pathophysiological changes include airway inflammation, airway hyperreactivity, and airway remodeling (Alberto et al., 2017). About 300 million people worldwide are reported to have asthma, causing about 1,000 deaths every day (GINA, 2025). In terms of pathological changes, airway inflammation is thought to be the core pathological link that triggers subsequent airway hyperresponsiveness, mucus secretion, and airway wall remodeling (Hamida and Bart, 2021). Eosinophils are considered to be an important effector cell in the development of allergic asthma. Extensive eosinophil infiltration around the airways is considered a hallmark of allergic asthma (Anuradha et al., 2016; Akira et al., 2020; Samuel et al., 2005).

During glycolysis, the main functional enzymes are hexokinase (HK), phosphofructokinase-1 (PFK-1), and pyruvate kinase (PK), as well as the production of glycolysis is lactate (Anoop Si et al., 2019). Notably, PFK-1 activity is controlled allosterically by fructose 2,6-bisphosphate (F2,6BP), whereas F2,6BP levels are controlled by 6-phosphofructo-2-kinase/fructose-2,6-bisphosphatase-3 (PFKFB3) (Sakakibara et al., 1997). Thus, PFKFB3 is a key enzyme regulating glycolysis (Mor et al., 2011). Current studies have identified glycolysis as a key player in the inflammatory response (Gonzalo et al., 2020). In parallel, recent studies have found that PFKFB3 is highly expressed in inflammatory cells and promotes activation of inflammatory cells through glycolysis (Linqiang et al., 2020; Yang et al., 2021). More studies have shown that PFKFB3 plays a key role in the inflammatory response. For example, increases in PFKFB3 were strongly associated with an exaggerated inflammatory response in sepsis (Xiao et al., 2023). 3PO inhibited lung inflammation and lung apoptosis in sepsis-induced acute lung injury in mice (Yuanqi et al., 2017). Inhibition of PFKFB3 was effective in reducing inflammatory responses induced by rheumatoid arthritis (Yaoyao et al., 2017). In addition, PFKFB3 was involved in the inflammatory response in diseases such as nephritis (Chengcheng et al., 2022), arthritis (Ting-Ting et al., 2025), and pancreatitis (Jing et al., 2025). PFKFB3 inhibitors such as 3PO, PFK015, and PFK158 can suppress the progression of various inflammatory diseases (Yinhu et al., 2020; Min et al., 2022; Haibing et al., 2019). Therefore, selective inhibition of PFKFB3 has the potential to be an important approach to suppress inflammatory diseases. At present, whether PFKFB3 regulates airway inflammation in asthma needs further study.

The nuclear factor kappa B (NF-κB) is involved in regulating cell proliferation, cell growth, inflammatory response, and immune response (Chen et al., 1999; Gius et al., 1999). NF-κB plays a central role in mediating inflammation. Following activation by inflammatory stimuli, a series of cascades occur, resulting in activation of IKB kinases (IKK), phosphorylation and degradation of inhibitor of nuclear factor Kappa B (IKB), followed by phosphorylation of NF-κB, thereby promoting its translocation from the cytoplasm to the nucleus. Finally, NF-κB amplifies the inflammatory response by regulating transcription and translation of pro-inflammatory mediators (Dominga et al., 2023). Dysregulation of NF-κB is associated with many inflammatory diseases, including rheumatoid arthritis, inflammatory bowel disease, multiple sclerosis, atherosclerosis, systemic lupus erythematosus, and type 2 diabetes (Hongmei et al., 2025). Several studies have shown that NF-κB is involved in regulating the inflammatory response process in asthma. For example, IFRD1 modulated asthmatic airway inflammation through the NF-κB pathway (Ming et al., 2020). Fibroblast growth factor 10 attenuated airway inflammation in allergic asthma by inhibiting the PI3K/AKT/NF-κB pathway (Wenjun et al., 2023). In addition, previous studies have shown that inhibition of PFKFB3 normalized inflammation in EC by blocking nuclear translocation of p65 protein and decreasing transcriptional activity of NF-κB signaling (such as p-NF-κB expression levels) (Wang et al., 2019; Anna Rita et al., 2016; Ruyuan et al., 2018; Wen et al., 2019).

Based on the above background, we hypothesize that pharmacological inhibition of PFKFB3 can attenuate airway inflammation in asthma, and that the underlying mechanism may involve the NF-κB signaling pathway. This investigation could reveal PFKFB3 as a promising novel therapeutic target for modulating inflammatory responses in asthma, thereby supporting the development of targeted therapies aimed at improved disease control and long-term clinical outcomes.

## 2 Materials and methods

### 2.1 Reagents

Enzyme-linked immunosorbent assay (ELISA) for interleukin-4 (IL-4), interleukin-5 (IL-5), interleukin-13 (IL-13), interleukin-17A (IL-17A), interleukin-1β (IL-1β), tumor necrosis factor-α (TNF-α), interleukin-6 (IL-6), Immunoglobulin E (IgE), chemokine (C-C motif) ligand 11 (CCL11), and interleukin-10 (IL-10) were purchased from JONLNBIO (Shanghai, China). The lactate assay kit was purchased from JONLNBIO (Shanghai, China). The primary antibodies were as follows: anti-chemokine (C-C motif) receptor 3 (CCR3) (22351-1-AP, Proteintech), anti-p-NF-κB p65 (TP56372, Abmart), anti-NF-κB p65 (T55034, Abmart), and anti-IKB-α (T55026, Abmart). The PFKFB3 inhibitor 3PO (HY-19824, MedChemExpress) was used in the experiments.

### 2.2 Potential targets of 3PO

The potential molecular targets of 3PO were predicted using SwissTargetPrediction (http://www.swisstargetprediction.ch/), Comparative Toxicogenomics Database (CTD) (https://ctdbase.org/), and GeneCards (https://www.genecards.org/) (accessed on 11 November 2025). Corresponding UniProt ID and gene names were retrieved from the UniProt database (https://www.uniprot.org/).

### 2.3 Identification of asthma-related gene targets

Collect asthma-related target genes from GeneCards (https://www.genecards.org), OMIM (http://omim.org/), and DrugBank (https://go.drugbank.com/) (accessed on 11 November 2025). After merging the data from the three databases, duplicates were removed; gene names were then retrieved from UniProt (https://www.uniprot.org/) to identify the disease-associated target genes.

### 2.4 Target prediction of 3PO against asthma

To identify overlapping genes, the predicted targets of 3PO and known asthma-related genes were submitted for analysis using the online Bioinformatics website (https://www.bioinformatics.com.cn/static/others/jvenn/example.html) (accessed on 11 November 2025). These intersecting genes were regarded as potential therapeutic targets of 3PO for the treatment of asthma.

### 2.5 Network analysis of protein-protein interaction (PPI)

The overlapping targets were imported into the STRING database (https://string-db.org/) to construct a PPI network. Network visualization and analysis were conducted using Cytoscape software (version 3.10.2) combined with Centiscape 2.2 plugins. To identify core genes, centrality parameters (Degree, Betweenness, and Closeness) were calculated for each node. Nodes with centrality values greater than the median of each respective parameter were selected as core nodes. The resulting core sub-network was then extracted for further analysis.

### 2.6 Enrichment analysis

Metascape (https://www.metascape.org/) was used for the Kyoto Encyclopedia of Genes and Genomes (KEGG) pathway and Gene Ontology (GO) enrichment analysis of common targets. The enrichment results were visualized with Bioinformatics (http://www.bioinformatics.com.cn/). The Benjamini–Hochberg (BH) method was used for multiple test correction to control the false discovery rate (FDR).

### 2.7 Molecular docking simulation

The small-molecule ligand 3PO and the core target-protein structures were retrieved from the PubChem database (https://pubchem.ncbi.nlm.nih.gov/) and the PDB database (https://www.rcsb.org/), respectively. The proteins were pretreated (water molecules removed, phosphate groups deleted, etc.) with PyMOL 3.0.3. Both the ligand and the proteins were then imported into CB-DOCK2 (https://cadd.labshare.cn/cb-dock2/php/index.php) for molecular docking, and the resulting complexes were visualized with PyMOL 3.0.3.

### 2.8 Molecular dynamics simulation

Molecular dynamics simulations of IL-1β-3PO, IL-6-3PO, TNF-α-3PO, and NF-κB p65-3PO complexes were performed using Gromacs 2025.2 software and AMBER99SB/ILDN force field to investigate the dynamic timing response of these complexes (David et al., 2005). The complex was placed in a cube box, and the distance between the edges of the box and the molecule was set to 1.0 nm. Under periodic boundary conditions, ion concentrations were adjusted by replacing part of the water molecules with Na^+^ and Cl^−^ ions at physiological concentrations (0.15 M). The steepest descent method was used to reduce the energy of the system to less than 1,000 kJ/(mol·nm). Subsequently, the system was pre-equilibrated under the NVT and NPT ensembles, respectively, followed by free molecular dynamics simulations.

The whole simulation process was performed for a total of 5,000,000 steps, with a step length of 2 fs and a total simulation time of 100 ns. After the simulation, the trajectories were analyzed using the Gromacs built-in tool to calculate the root mean square deviation (RMSD), root mean square fluctuation (RMSF), and protein radius of gyration (Rg) parameters of each amino acid trajectory, and further calculate the free energy (MM/PBSA), free energy distribution map, and other related data.

### 2.9 Experimental animals and methods

Specific-pathogen-free (SPF) BALB/c male mice (6–8 weeks old, 20–25 g) were used in this study. All experiments involving animals were approved by the Institutional Animal Ethics Committee of HUA TENG (approval number: HTSW210718). All male BALB/c mice were acclimated to the standard animal laboratory environment for 1 week before the start of the experiment. According to our previous study (Deng et al., 2020), OVA-induced asthma mouse models were established. Mice were randomly assigned to three groups (n = 5 per group) for the sensitization, challenge, and treatment protocol. The first group was the non-sensitized control group; the second group was the OVA group; and the third group received OVA + 3PO.

The sensitization process was carried out by intraperitoneal injection of the mixture of ovalbumin (OVA) and aluminium hydroxide on days 1 and 7. The OVA challenge was administered via intranasal administration of OVA on days 14–16 and 21–23. 3PO treatment was administered intraperitoneally at 25 mg/kg on days 14–16 and 21–23.

After anesthesia via intraperitoneal injection of pentobarbital sodium (50 mg/kg), the mice were euthanized by exsanguination to collect bronchoalveolar lavage fluid (BALF) and lung tissues. BALF was collected for ELISA and total cell count. The right lung was stored in liquid nitrogen, and the left lung was fixed in 4% paraformaldehyde.

### 2.10 Analysis of BALF

After collection and total cell counting, BALF samples were centrifuged at 1,000 rpm for 10 min to separate the supernatant, which was then stored at −80 °C. Concentrations of IL-4, IL-5, IL-13, IL-17A, IL-1β, TNF-α, IL-6, lactate, IgE, CCL11, and IL-10 in the BALF supernatant were determined using ELISA kits and a lactate assay kit, according to the manufacturer’s instructions.

### 2.11 Histological and immunostaining

The left lung of mice was excised and fixed in paraffin. Lung sections (4 μm) were prepared and stained with H&E for histopathological assessment. Periodic acid-Schiff (PAS) staining was performed to assess mucus production and goblet cell hyperplasia.

For immunohistochemistry (IHC) and immunofluorescence (IF), lung sections were deparaffinized, rehydrated, and subjected to antigen retrieval by heating in Tris-EDTA buffer (pH 9.0).

For IHC detection of PFKFB3, endogenous peroxidase activity was blocked with 3% H_2_O_2_ for 10 min at room temperature. After blocking with goat serum for 1 h at room temperature, the sections were incubated overnight at 4 °C with an anti-PFKFB3 antibody. Following washing with PBS, the sections were incubated with a horseradish peroxidase (HRP)-conjugated secondary antibody for 1 h at room temperature. Immunoreactivity was visualized using a diaminobenzidine (DAB) substrate kit, and the sections were counterstained with hematoxylin. Images were captured using a light microscope.

For IF detection of CCR3, after antigen retrieval, sections were blocked with goat serum for 1 h at room temperature and then incubated overnight at 4 °C with an anti-CCR3 antibody. Following washing with PBS, the sections were incubated with a fluorescently labeled goat anti-rabbit IgG (H + L) secondary antibody for 1 h at room temperature in the dark. Cell nuclei were identified by DAPI staining. Finally, tissues were imaged with a fluorescence microscope.

### 2.12 Western blotting analysis

Tissue lysates from the right lungs of mice were collected, denatured by boiling in SDS sample buffer, and subjected to western blotting to detect the following proteins: p-NF-κB p65, NF-κB p65, and IKB-α. After incubation with a secondary antibody, immunoreactive bands were visualized using an imaging system. Band densities were quantified, normalized to β-actin, and expressed as fold changes relative to the control group.

### 2.13 Statistical analysis

Statistical analysis was performed using GraphPad Prism 9. All data are expressed as the mean ± SEM. Comparisons between two groups were performed using Student’s t-test. Comparisons among multiple groups were conducted by one-way analysis of variance (ANOVA), followed by Tukey’s post-hoc test for multiple comparisons when ANOVA revealed significant differences. Statistical significance was set at p < 0.05.

## 3 Results

### 3.1 Acquisition of common target genes for 3PO and asthma

**FIGURE 1:**
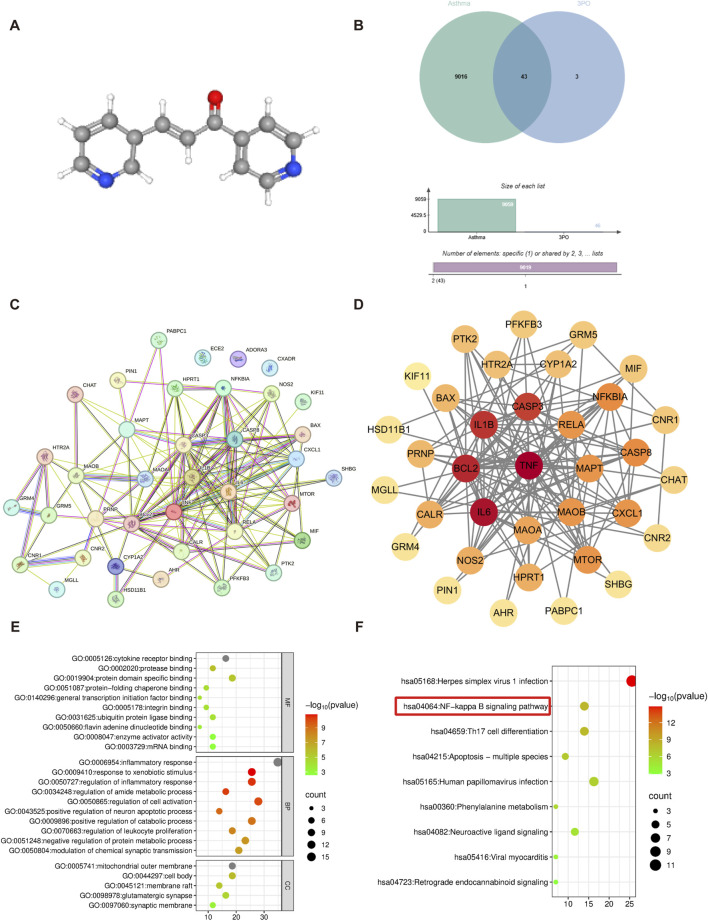
Network pharmacology analysis of the PFKFB3 inhibitor 3PO’s potential anti-asthma targets. **(A)** The chemical structure of 3PO. **(B)** Venn diagram illustrating 43 overlapping targets between 3PO-predicted targets and asthma-related genes. **(C)** PPI network constructed from the 43 intersecting targets. **(D)** Top 9 hub genes identified and visualized in Cytoscape based on degree centrality: node color intensity (red) corresponds to higher connectivity. **(E)** GO enrichment analysis results, including BP, CC, and MF. **(F)** KEGG enrichment analysis results. Protein-protein interaction = PPI, GO = Gene ontology, BP = Biological process, CC = Cellular component, MF = Molecular function, KEGG = Kyoto encyclopedia of genes and genomes.

The chemical structure of 3PO is depicted in Figure 1A. From GeneCards, OMIM, and DrugBank, we initially collected 8986, 333, and 114 asthma-related targets, respectively (9433 in total); after duplicate removal, 9059 unique targets remained. For 3PO, SwissTargetPrediction, CTD, and GeneCards yielded 24, 9, and 16 targets, respectively (49 in total), which collapsed to 46 unique targets after deduplication. The intersection between the 3PO targets and the asthma-related genes was then generated with the online bioinformatics platform Venn, revealing 43 overlapping targets (Figure 1B). These 43 genes represent the candidate targets potentially mediating the therapeutic effects of 3PO in asthma.

### 3.2 PPI network construction and core target gene screening

To further investigate the interactions among these overlapping targets, the 43 genes were input into the STRING database to construct a PPI network (Figure 1C). The PPI network was subsequently visualized with Cytoscape 3.10.2, yielding 35 nodes and 144 edges. Centrality parameters (Degree, Closeness, and Betweenness) were calculated with the CentiScape 2.2 plugin. Using the median values of each centrality parameter as thresholds (Degree ≥8.229, Betweenness ≥35.714, and Closeness ≥0.015), we identified a core sub-network consisting of 9 nodes and 31 edges. As shown in Figure 1D and Table 1, the genes identified were TNF, IL6, BCL2, IL1B, CASP3, RELA, MAPT, MAOB, and MAOA. These nine genes, which include key inflammatory mediators (TNF, IL6, IL1B) and the NF-κB subunit RELA, were prioritized as core targets for further investigation, suggesting that 3PO may modulate asthma inflammation through these central nodes.

**TABLE 1:** Top 9 targets of 3PO against asthma.

Number	Gene Symbol	Gene Full Name
1	TNF	Tumor necrosis factor
2	IL6	Interleukin 6
3	BCL2	BCL2 apoptosis regulator
4	IL1B	Interleukin 1 beta
5	CASP3	Caspase 3
6	RELA	RELA Proto-oncogene, NF-κB subunit
7	MAPT	Microtubule associated protein Tau
8	MAOB	Monoamine oxidase B
9	MAOA	Monoamine oxidase A

### 3.3 GO and KEGG pathway enrichment analysis

To further clarify the biological functions of the 43 overlapping targets through which 3PO acts against asthma, GO and KEGG enrichment analyses were performed with Metascape. In total, 607 GO terms were retrieved: 551 biological process (BP) terms, 21 cellular component (CC) terms, and 35 molecular function (MF) terms, all filtered at P ≤ 0.05. As shown in Figure 1E, the top BP categories included inflammatory response, response to xenobiotic stimulus, regulation of inflammatory response, regulation of amide metabolic process, regulation of cell activation, and positive regulation of neuron apoptotic process. CC analysis indicated that 3PO mainly localizes to the mitochondrial outer membrane, cell body, membrane raft, glutamatergic synapse, and synaptic membrane. MF enrichment revealed significant involvement in cytokine receptor binding, protease binding, protein domain-specific binding, protein-folding chaperone binding, general transcription initiation factor binding, and integrin binding.

Enrichment analysis of KEGG pathways revealed 105 associated pathways. Of these, the nine most statistically significant pathways (p-value ranked) were selected for visual representation in a bubble plot (Figure 1F). The identified pathways, encompassing herpes simplex virus 1 infection, NF-κB signaling pathway, Th17 cell differentiation, apoptosis-multiple species, human papillomavirus infection, and phenylalanine metabolism, collectively indicate a potential mechanistic basis for the anti-asthmatic activity of 3PO. Notably, the NF-κB signaling pathway, which is central to asthmatic inflammation as previously established (Ming et al., 2020; Wenjun et al., 2023), was significantly enriched in our analysis. This finding indicates that 3PO may exert its anti-asthmatic effects through modulation of the NF-κB pathway.

### 3.4 Molecular docking simulation

**FIGURE 2:**
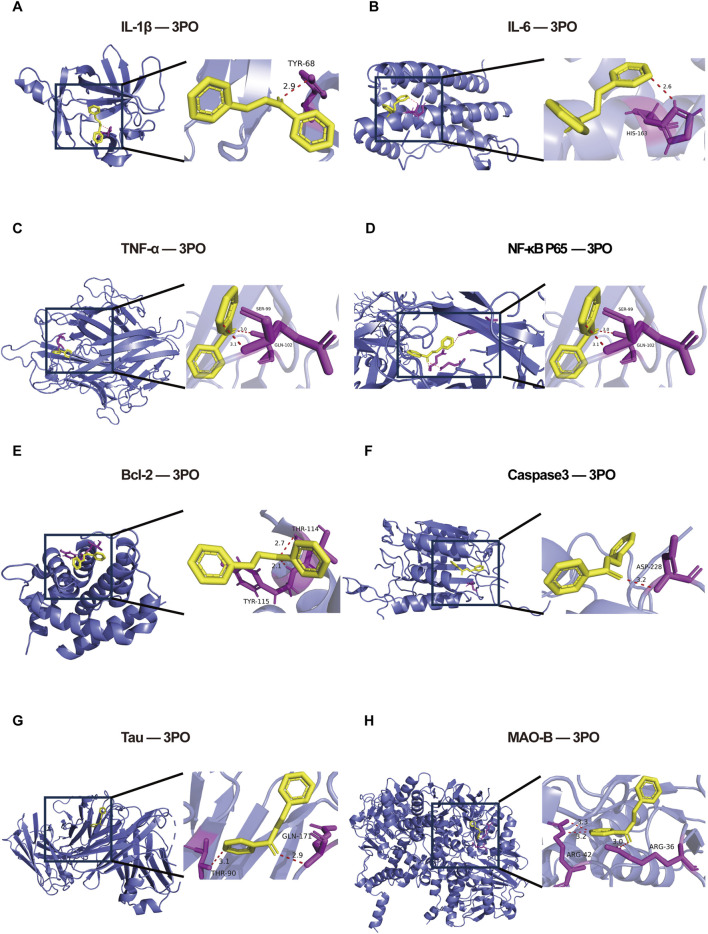
Molecular docking results of 3PO with the top eight core targets. **(A)** Molecular docking of 3PO with IL-1β. **(B)** Molecular docking of 3PO with IL-6. **(C)** Molecular docking of 3PO with TNF-α. **(D)** Molecular docking of 3PO with NF-κB p65. **(E)** Molecular docking of 3PO with Bcl-2. **(F)** Molecular docking of 3PO with Caspase3. **(G)** Molecular docking of 3PO with Tau. **(H)** Molecular docking of 3PO with MAO-B. IL-1β = Interleukin-1β, IL-6 = Interleukin-6, TNF-α = Tumor necrosis factor-α, NF-κB = Nuclear factor kappa B.

Molecular docking simulations reliably predict how—and how tightly—a small molecule binds to its target protein, offering an early gauge of both potency and selectivity for candidate compounds (Douglas et al., 2004). Inversely correlated with binding affinity, lower docking scores suggest stronger binding. Scores below −5.0 kcal/mol typically indicate potential interactions, while those below −7.0 kcal/mol are regarded as indicative of robust binding. As listed in Table 2 and illustrated in Figures 2A–H, the docking scores of 3PO with IL-1β, IL-6, TNF-α, NF-κB p65, Bcl-2, Caspase3, Tau, and MAO-B are −5.9, −6.0, −6.9, −5.9, −6.1, −6.1, −7.0, and −8.5 kcal/mol, respectively. Molecular docking for MAO-A was not performed, as a high-quality human MAO-A crystal structure was not available in the PDB database at the time of this study.

**TABLE 2:** The binding energy values of core compounds of 3PO and core targets.

Targets	PDB	Binding enery (kcal/mol)
IL-1β	5R8Q	−5.9
IL-6	7NXZ	−6.0
TNF-α	1TNF	−6.9
NF-κB p65	3GUT	−5.9
Bcl-2	5UUK	−6.1
Caspase-3	5IBP	−6.1
Tau	6PXR	−7.0
MAO-B	2XCG	−8.5
MAO-A	-	-

### 3.5 Molecular dynamics simulation

Molecular dynamics simulations provide a more accurate assessment of protein-ligand binding stability and conformational changes. They can also reveal intricate details beyond the reach of experimental methods, thereby enabling a deeper exploration of the underlying mechanism of action (Ananta et al., 2022). According to previous studies, IL-1β, IL-6, TNF-α, and NF-κB p65 play significant roles in the inflammatory response of asthma. Therefore, to explain the interaction between 3PO and IL-1β, IL-6, TNF-α, and NF-κB p65, we carried out a molecular dynamics simulation with a duration of 100 ns.

**FIGURE 3:**
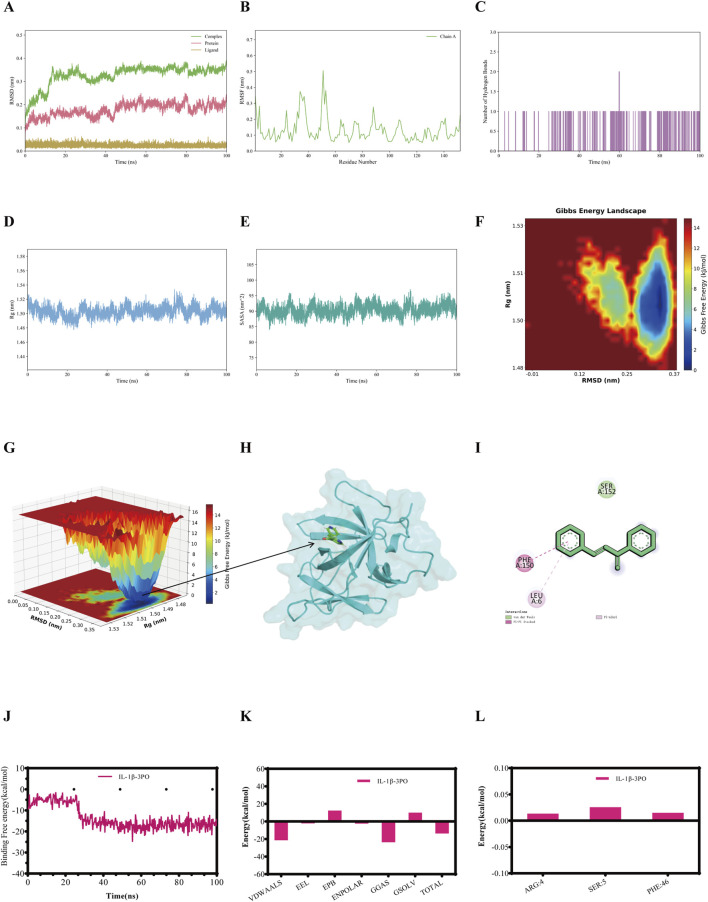
The molecular dynamics simulations profiles of the IL-1β-3PO complex. **(A)** RMSD values (Complex: IL-1β-3PO, Protein: IL-1β, Ligand: 3PO). **(B)** RMSF analysis. **(C)** Number of hydrogen bonds formed between IL-1β and 3PO during the simulation. **(D)** Rg analysis. **(E)** SASA analysis. **(F)** Gibbs free energy landscape (2D). **(G)** Gibbs free energy landscape (3D). **(H)** The lowest energy 3D conformation. **(I)** 2D patterns of bonds at the most stable condition of the complex. **(J)** Time evolution of the MM/GBSA binding free energy. **(K)** Decomposition of the MM/GBSA binding free energy. **(L)** Per-residue energy decomposition. RMSD = Root mean square deviation, RMSF = Root mean square fluctuation, Rg = Radius of gyration, SASA = Solvent accessible surface area.

**FIGURE 4:**
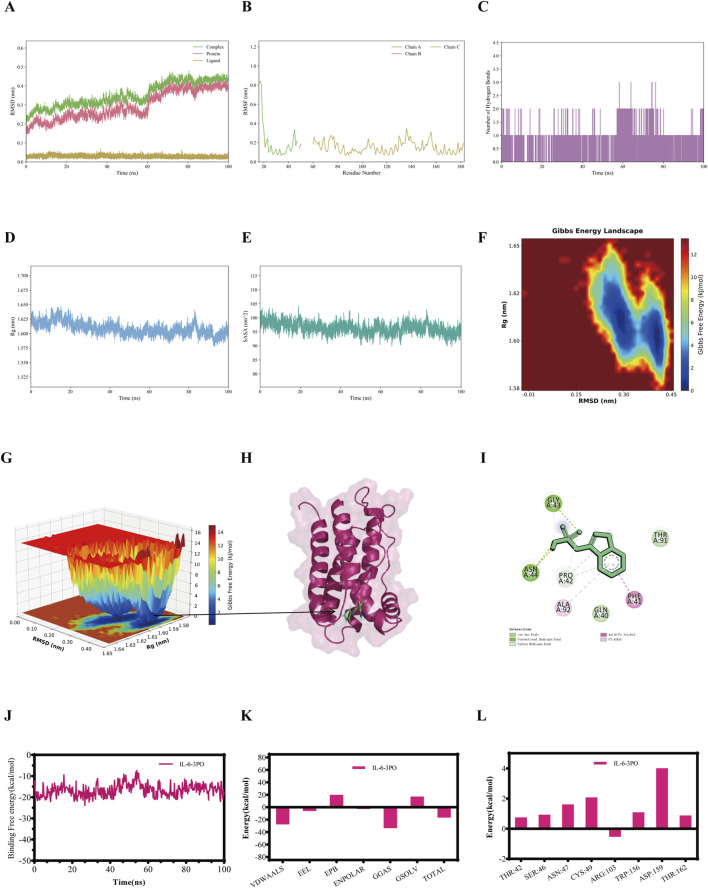
The molecular dynamics simulations profiles of the IL-6-3PO complex. **(A)** RMSD values (Complex: IL-6-3PO, Protein: IL-6, Ligand: 3PO). **(B)** RMSF analysis. **(C)** Number of hydrogen bonds formed between IL-6 and 3PO during the simulation. **(D)** Rg analysis. **(E)** SASA analysis. **(F)** Gibbs free energy landscape (2D). **(G)** Gibbs free energy landscape (3D). **(H)** The lowest energy 3D conformation. **(I)** 2D patterns of bonds at the most stable condition of the complex. **(J)** Time evolution of the MM/GBSA binding free energy. **(K)** Decomposition of the MM/GBSA binding free energy. **(L)** Per-residue energy decomposition.

**FIGURE 5:**
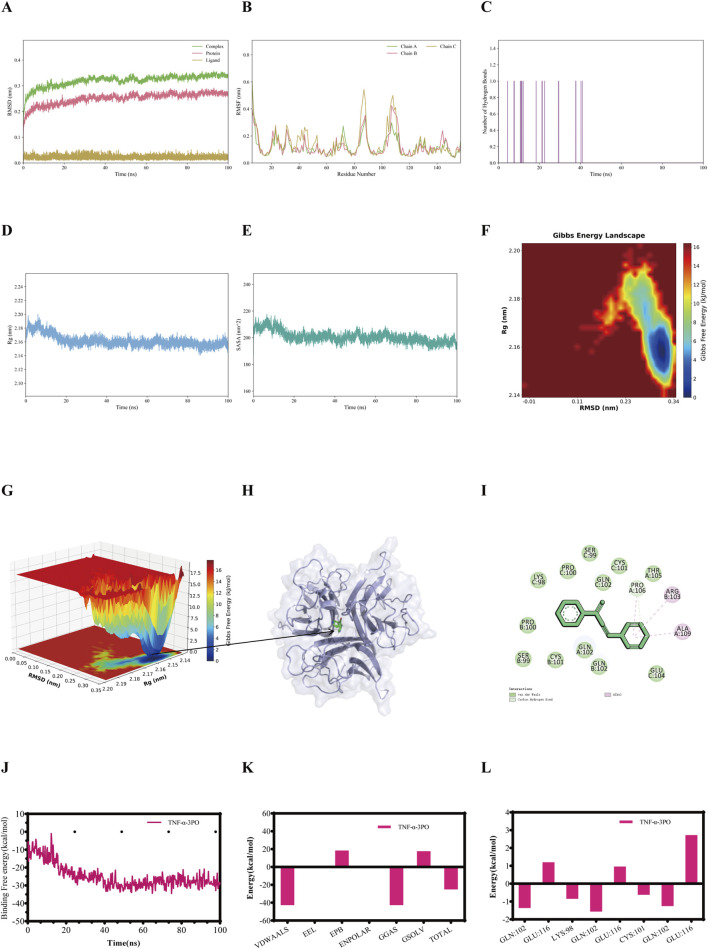
The molecular dynamics simulations profiles of the TNF-α-3PO complex. **(A)** RMSD values (Complex: TNF-α-3PO, Protein: TNF-α, Ligand: 3PO). **(B)** RMSF analysis. **(C)** Number of hydrogen bonds formed between TNF-α and 3PO during the simulation. **(D)** Rg analysis. **(E)** SASA analysis. **(F)** Gibbs free energy landscape (2D). **(G)** Gibbs free energy landscape (3D). **(H)** The lowest energy 3D conformation. **(I)** 2D patterns of bonds at the most stable condition of the complex. **(J)** Time evolution of the MM/GBSA binding free energy. **(K)** Decomposition of the MM/GBSA binding free energy. **(L)** Per-residue energy decomposition.

**FIGURE 6:**
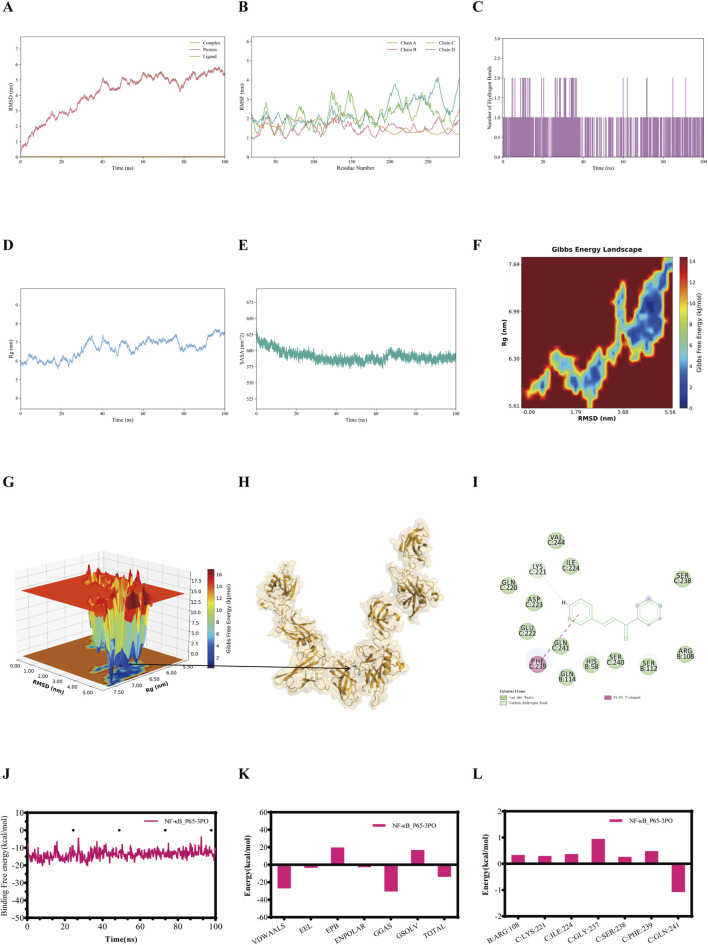
The molecular dynamics simulations profiles of NF-κB p65-3PO complex. **(A)** RMSD values (Complex: NF-κB p65-3PO, Protein: NF-κB p65 Ligand: 3PO). **(B)** RMSF analysis. **(C)** Number of hydrogen bonds formed between NF-κB p65 and 3PO during the simulation. **(D)** Rg analysis. **(E)** SASA analysis. **(F)** Gibbs free energy landscape (2D). **(G)** Gibbs free energy landscape (3D). **(H)** The lowest energy 3D conformation. **(I)** 2D patterns of bonds at the most stable condition of the complex. **(J)** Time evolution of the MM/GBSA binding free energy. **(K)** Decomposition of the MM/GBSA binding free energy. **(L)** Per-residue energy decomposition.

RMSD was employed to assess the conformational differences and trajectory stability of proteins and small molecules during the dynamic binding process. Following an initial period, the RMSD value of the IL-1β-3PO, IL-6-3PO, TNF-α-3PO, and NF-κB p65-3PO complexes stabilized within 100 ns (Figures 3A, 4A, 5A, 6A). The stability profiles of these complexes were comparable to those of the respective unliganded proteins, suggesting that 3PO may form relatively stable associations with these targets under the simulated conditions (Morteza et al., 2023).

RMSF quantifies the positional fluctuation of individual atoms during a simulation and serves as a direct indicator of local flexibility within the molecular structure (Ting et al., 2022). RMSF peaks highlight flexible regions, such as loops or active-site-adjacent areas, that are potentially involved in substrate interaction. Their increased mobility likely facilitates ligand interaction and conformational changes, thus possibly enabling functional adaptability. In contrast, lower RMSF values indicate rigid, structurally stable segments that are crucial for maintaining the overall structural integrity of the complex. The fluctuation amplitudes of the peptide residues in the IL-1β-3PO, IL-6-3PO, TNF-α-3PO, and NF-κB p65-3PO complexes are shown, respectively, in Figures 3B, 4B, 5B, 6B.

Hydrogen bonds serve as an indicator for assessing the interaction forces between the small molecules and the protein. The hydrogen bonds exhibit continuous fluctuations, engaging in a dynamic exchange throughout the entire trajectory, indicating that there is an instantaneous but persistent interaction between the small molecules and the protein. During the 100 ns simulation, the IL-1β-3PO, IL-6-3PO, TNF-α-3PO, and NF-κB p65-3PO complexes had hydrogen bond numbers ranging from 1–2, 1–3, 0–1, and 1-2, respectively, as shown in Figures 3C, 4C, 5C, 6C.

The stable Rg value indicates that the complex maintains a relatively consistent tertiary structure, without any significant expansion or contraction, suggesting the stability of its structure. A lower Rg value corresponds to a more compact conformation. During the 100 ns simulations, the Rg values stabilized at 1.50–1.52nm, 1.60–1.65nm, 2.16–2.18nm, and 6–7nm for IL-1β-3PO, IL-6-3PO, TNF-α-3PO, and NF-κB p65-3PO complexes, respectively (Figures 3D, 4D, 5D, 6D).

Solvent accessible surface area (SASA) serves as a key metric to quantify changes in protein surface area, providing insights into the folding stability and conformational compactness of the complex. The SASA values for IL-1β-3PO, IL-6-3PO, TNF-α-3PO, and NF-κB p65-3PO complexes remained stable throughout the simulation, suggesting that 3PO binding maintains the size and solvent accessibility of the protein binding pocket (Figures 3E, 4E, 5E, 6E).

Gibbs free energy landscape employs principal component analysis to assess the conformational space of the complex samples. It is constructed using RMSD and Rg as coordinates. The blue areas represent low-energy conformational states, indicating stable configurations, while the red areas represent high-energy states. Gibbs free energy profile of the IL-1β-3PO, IL-6-3PO, TNF-α-3PO, and NF-κB p65-3PO complexes showed a clear minimum energy well, respectively (Figures 3F,G, 4F,G, 5F,G, as well as Figures 6F,G). In addition, the lowest energy 3D conformation of the IL-1β-3PO, IL-6-3PO, TNF-α-3PO, and NF-κB p65-3PO complexes are shown in the Figures 3H, 4H, 5H, 6H, respectively. The 2D interaction diagram of the IL-1β-3PO, IL-6-3PO, TNF-α-3PO, and NF-κB p65-3PO complexes (Figures 3I, 4I, 5I, 6I) revealed the key molecular contacts at the most stable condition.

To identify the key energetic drivers in the IL-1β-3PO, IL-6-3PO, TNF-α-3PO, and NF-κB p65-3PO dynamic interactions, we carried out the MM/GBSA method to compute the binding free energy (Junkun et al., 2024). In addition, binding free energy data for the IL-1β-3PO, IL-6-3PO, TNF-α-3PO, and NF-κB p65-3PO complexes are presented in the Figures 3J–L, 4J–L, 5J–L, 6J–L, respectively. When the total binding free energy is negative, the larger the absolute value, the stronger the constraint. The total binding free energy of the IL-1β-3PO, IL-6-3PO, TNF-α-3PO, and NF-κB p65-3PO complexes is negative, indicating good binding in these complexes.

In summary, the 100 ns molecular dynamics simulations, consistent with the molecular docking outcomes, suggest possible interactions between 3PO and IL-1β, IL-6, TNF-α, and NF-κB p65. These *in silico* findings provide a theoretical basis for a potential role of 3PO in modulating asthma-related inflammatory reactions, warranting further experimental validation.

### 3.6 3PO inhibited the airway inflammation in the OVA-induced asthma mouse models

**FIGURE 7:**
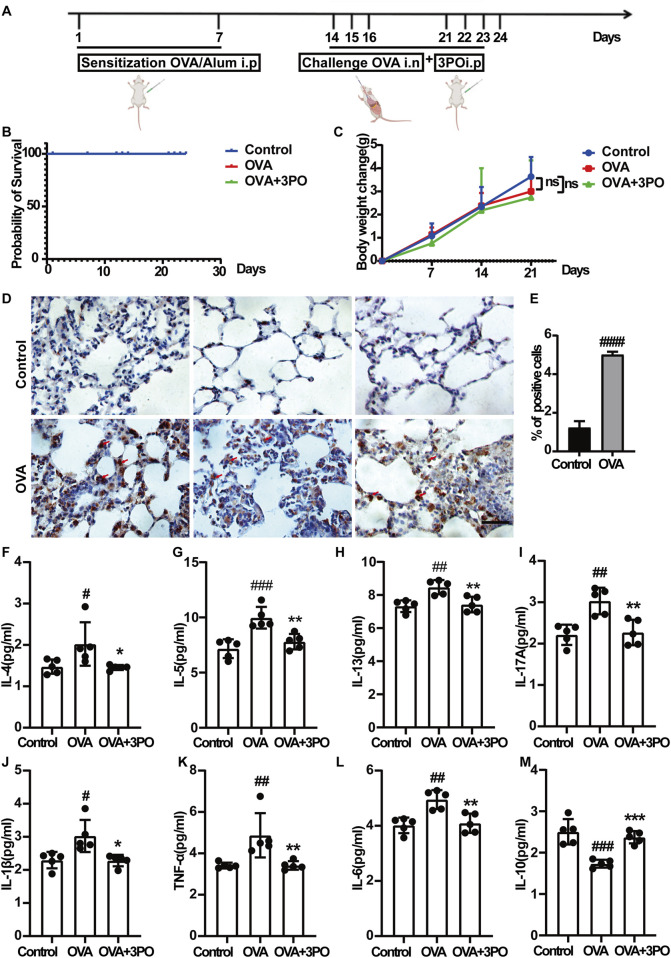
PFKFB3 inhibitor 3PO attenuated airway inflammation in OVA-induced asthmatic mice. **(A)** A schematic diagram of the design of animal experiments. Mice were sensitized and challenged with OVA, followed by treatment with 3PO. **(B)** Survival rate of mice during the experiment (no mortality observed). **(C)** Body weight changes of mice throughout the protocol. **(D)** PFKFB3 expression in lung sections was detected by immunohistochemistry. Red arrows indicate PFKFB3-positive cells. Scale bar = 50 μm. **(E)** Quantitative analysis of PFKFB3-positive cells in lung tissues. **(F–M)** Levels of cytokines in BALF, including **(F)** IL-4, **(G)** IL-5, **(H)** IL-13, **(I)** IL-17A, **(J)** IL-1β, **(K)** TNF-α, **(L)** IL-6, and **(M)** IL-10. Data are presented as the mean ± SD (n = 5 per group). #P < 0.05, ##P < 0.01, ###P < 0.001, ####P < 0.0001 vs. the control group. *p < 0.05, **p < 0.01, ***p < 0.001 vs. the OVA group. BALF = Bronchoalveolar lavage fluid. IL-4 = Interleukin-4, IL-5 = Interleukin-5, IL-13 = Interleukin-13, IL-17A = Interleukin-17A, IL-10 = Interleukin-10.

The study protocol of the animal experiment is shown in Figure 7A. Mice treated with 3PO had normal survival and no mortality during the study period (Figure 7B). We monitored the weight change of mice in each group throughout the experiment. As shown in Figure 7C, the body weight of mice in the normal control group, OVA asthma model group, and each treatment group showed a steady trend of increase, and the body weight change curves basically coincided between the groups.

**FIGURE 8:**
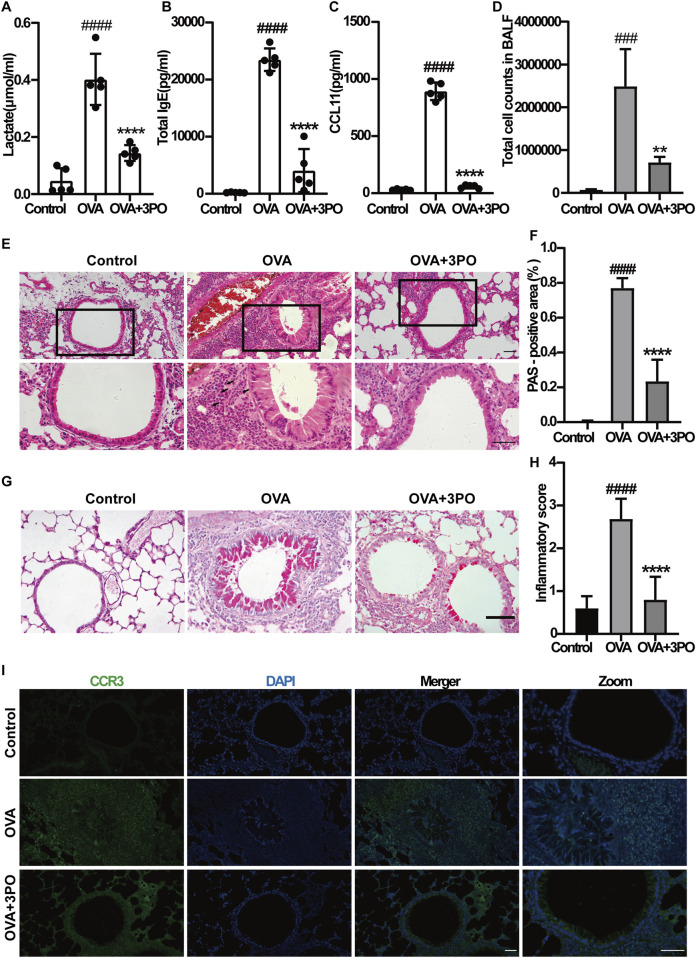
PFKFB3 inhibitor 3PO attenuated airway inflammation in OVA-induced asthmatic mice. **(A)** Lactate levels, **(B)** IgE levels, and **(C)** CCL11 levels in BALF. **(D)** Total cell counts in BALF. **(E)** Representative images of H&E staining of lung tissue sections. Black arrows indicate eosinophils. Magnification: ×200 (upper panel) and 400× (lower panel). Scale bar = 50 μm. **(F)** Quantitative analysis of PAS-positive area in lung tissues. **(G)** Airway mucus secretion and goblet cell hyperplasia were detected using PAS staining. Scale bar = 50 μm. **(H)** Quantitative inflammatory cell infiltration scores. **(I)** Representative immunofluorescence staining for CCR3 (green) in bronchial regions; nuclei were stained with DAPI (blue). Magnification: ×200 (left panel) and 400× (right panel). Scale bar = 50 μm. Data are presented as the mean ± SD (n = 5 per group). ###P < 0.001, ####P < 0.0001 vs. the control group. **p < 0.01, ****p < 0.0001 vs. the OVA group. IgE = Immunoglobulin E, CCR3 = Chemokine (C-C motif) receptor 3, H&E = Hematoxylin and eosin, PAS = Periodic acid-Schiff, CCL11 = Chemokine (C-C motif) ligand 11.

To assess PFKFB3 expression in the lung, we performed IHC. As shown in Figures 7D,E, its levels were markedly increased in the lung tissues of OVA-induced asthmatic mice compared to controls. Notably, this upregulation suggests that PFKFB3 contributes to asthma pathology, rationalizing the use of its inhibitor 3PO. We then examined the effects of 3PO on inflammatory mediators in BALF. As shown in Figures 7F–M, 8A–D, IL-4, IL-5, IL-13, IL-17A, IL-1β, TNF-α, IL-6, lactate, IgE, CCL11, and the total cell number were increased, while IL-10 was decreased in the BALF of OVA-induced asthmatic mice, and these effects were alleviated by 3PO treatment.

Histological analysis of lung tissue showed that significant lung lesions, significant airway epithelial hyperplasia, significant tracheal stenosis, airway smooth muscle layer thickening, and significantly increased vascular and branch airway inflammatory cell infiltration, especially eosinophils, occurred in the lung tissue of mice in the OVA group compared with the control group, but they could be alleviated by 3PO treatment (Figure 8E). To further evaluate the effect of 3PO on airway mucus secretion and goblet cell hyperplasia, we performed PAS staining. Compared with the control group, lung tissues from OVA-induced asthmatic mice exhibited significant goblet cell hyperplasia and excessive mucus production, as indicated by a substantial increase in PAS-positive staining area. Notably, administration of 3PO markedly reduced the PAS-positive area, suggesting an alleviation of airway mucus secretion and goblet cell hyperplasia (Figures 8F,G).

In addition, the degree of inflammation was scored using the airway inflammatory cell infiltration score scale, and mice in the OVA asthma group had significantly increased inflammation scores compared with the control group, while they were significantly reduced after 3PO treatment (Figure 8H). To further observe the eosinophils beside the branch airways, immunofluorescence staining for CCR3 was performed, and it was found that indeed 3PO could reduce the increase of eosinophils beside the branch airways in OVA-induced asthmatic mice (Figure 8I). These results suggest that 3PO, an inhibitor of PFKFB3, attenuates airway inflammation in asthmatic mice.

### 3.7 3PO inhibited the NF-κB pathway activation in the OVA-induced asthma mouse models

**FIGURE 9:**
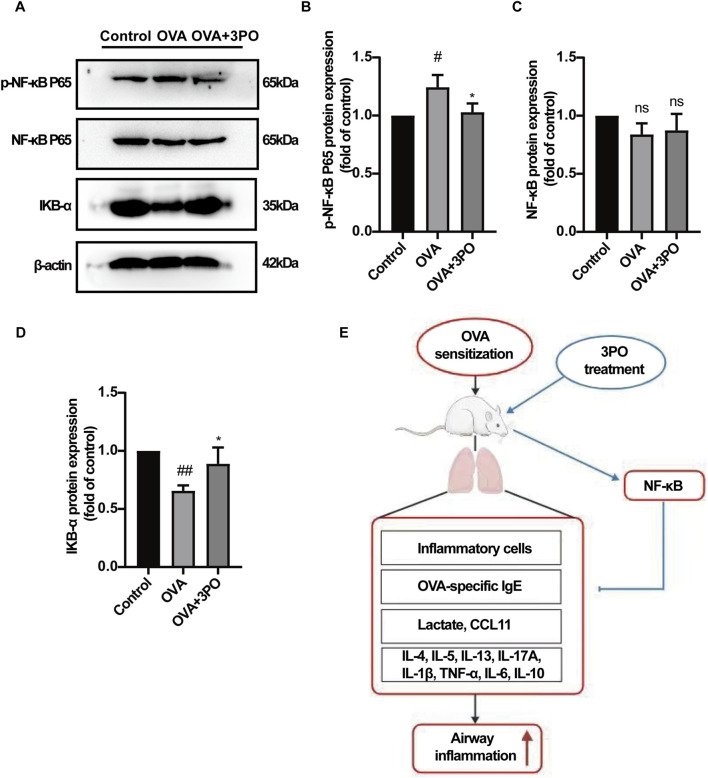
PFKFB3 inhibitor 3PO inhibited the NF-κB signaling pathway activation in the OVA-induced asthma mouse models. **(A)** Western blot analysis of p-NF-κB P65, NF-κB P65, IKB, and β-actin protein expression. **(B–D)** Quantitative analysis of **(B)** p-NF-κB P65, **(C)** total NF-κB P65, and **(D)** IKB protein expression, normalized to β-actin. **(E)** Schematic diagram illustrating the effect of 3PO on OVA-induced airway inflammation in asthma. Data are presented as the mean ± SD. #P < 0.05, ##P < 0.01 vs. the control group. *p < 0.05 vs. the OVA group. ns = no significance. IKB = Inhibitor of nuclear factor kappa B.

To elucidate the anti-inflammatory mechanism of 3PO suggested by the above bioinformatics analysis, we examined key proteins in the NF-κB pathway. In OVA-induced mice, p-NF-κB P65 levels were elevated compared to controls, an effect that was reversed by 3PO treatment (Figures 9A,B). Conversely, total NF-κB P65 protein levels were not significantly altered across groups (Figures 9A,C). Moreover, 3PO rescued the OVA-induced decrease in IKB-α protein levels (Figures 9A,D). Collectively, these results indicated that 3PO exerts its therapeutic effect in allergic asthma associated with the suppression of the NF-κB pathway activation.

## 4 Discussion

An integrative methodology, incorporating network pharmacology, molecular docking, molecular dynamics simulations, and *in vivo* experiments, was implemented to probe the effects of 3PO in asthma. Our research demonstrated two principal findings. Firstly, 3PO exerts an anti-inflammatory effect on asthma. Secondly, we found that the anti-inflammatory action of 3PO in asthma may involve suppression of the NF-κB signaling pathway. Our findings provide a basis for future research and highlight its therapeutic potential.

Network pharmacology provides a systematic approach to identify drug and disease targets (Cristian et al., 2021). Initially, overlapping targets were identified by intersecting the 46 predicted targets of 3PO with 9,059 asthma-related genes. Subsequently, a PPI network was constructed, revealing nine hub genes (TNF, IL6, BCL2, IL1B, CASP3, RELA, MAPT, MAOB, and MAOA). These genes are primarily involved in signal transduction, inflammation, and apoptosis. KEGG pathway enrichment analysis notably highlighted the NF-κB signaling pathway, a pivotal regulator of inflammation (Barnes and Karin, 1997).

Molecular docking and molecular dynamics simulation studies can analyze key interaction mechanisms and evaluate the conformational stability of the resulting complexes (Anamika et al., 2020). Molecular docking suggested potential binding of 3PO to IL-1β, IL-6, TNF-α, NF-κB p65, Bcl-2, Caspase3, Tau, and MAO-B. Subsequently, molecular dynamics simulations were conducted to characterize the dynamic stability of these protein-ligand complexes (IL-1β/IL-6/TNF-α/NF-κB p65-3PO). Molecular dynamics simulation results found that these complexes formed after 3PO binding showed similar trends in terms of conformational stability, protein tightness, and wave behavior. The results of time-dependent RMSD, Rg, SASA, the number of Hydrogen bonds, as well as binding free energy, and RMSF analysis indicated that both systems achieve equilibrium. Collectively, these computational findings raise the possibility that 3PO may exert its effects in asthma through potential interactions with these inflammatory mediators, although this hypothesis requires confirmation by biochemical assays.

Previous studies have shown that PFKFB3 affects inflammatory responses through various mechanisms (Yixin et al., 2024; Xiaodong et al., 2025; Yue et al., 2024), and its inhibitor 3PO has anti-inflammatory effects (Wen et al., 2019; Adriana Fernanda et al., 2024). However, it is currently unclear whether PFKFB3 is involved in modulating airway inflammation during asthma, necessitating additional research. Therefore, we conducted *in vivo* validation experiments using the OVA-induced asthma mouse models. We first observed that PFKFB3 expression was upregulated in the lung tissues of asthmatic mice. 3PO treatment alleviated key pathological features of asthma in this model, such as airway mucus hypersecretion, goblet cell hyperplasia, and peribronchial infiltration of inflammatory cells, especially eosinophils. Furthermore, the upregulation of CCL11 (a CCR3 ligand critical for eosinophil recruitment) in the OVA-induced asthma mouse models was attenuated by treatment with 3PO. Eosinophils play a crucial role in the pathogenesis of bronchial asthma, which is widely recognized (Bousquet et al., 1990).

Moreover, the 3PO treatment reversed the dysregulated mediators in the BALF of asthmatic mice by lowering the levels of IL-4, IL-5, IL-13, IL-17A, IL-1β, TNF-α, IL-6, and IgE, which are factors related to asthma inflammation, and elevating the levels of IL-10. IL-10 is an anti-inflammatory cytokine that can inhibit pro-inflammatory cytokines (De Waal Malefyt et al., 1991). IL-10 levels are decreased in asthma patients versus healthy controls (Sareh et al., 2014), and this insufficient response is linked to persistent disease (Kandane-Rath et al., 2013). Furthermore, exogenous IL-10 has been shown to inhibit airway inflammation and hyperresponsiveness in mouse models of asthma (Masaya et al., 2022). Meanwhile, we observed a marked accumulation of lactate, the end-product of glycolysis, in the BALF of asthmatic mice, which was reversed following 3PO administration in accordance with improved inflammatory parameters. Importantly, this anti-inflammatory effect was achieved without adversely affecting animal survival or body weight, underscoring a favorable safety profile within the experimental context.

Furthermore, our *in vivo* results were consistent with the bioinformatics prediction, suggesting that 3PO treatment was associated with suppressed NF-κB pathway activation, as evidenced by reduced phosphorylation of NF-κB p65 and restored expression of its inhibitor, IKB-α. This is significant because the NF-κB pathway is a master regulator of inflammatory response in asthma (Soon-Young et al., 2024). For instance, probiotics and prebiotics have been found to attenuate airway inflammation in OVA-LPS-induced allergic asthma by modulating the TLR4/NF-kB signaling pathway (Zhiwei et al., 2022).

Several limitations of this study should be acknowledged. First, while molecular docking and molecular dynamics simulation are valuable predictive tools, the identified interactions between 3PO and its potential targets remain computational and require experimental validation. Second, measurements of AHR and lung function were not obtained, as our experimental setup at the time was not equipped for these assessments. Third, specific NF-κB inhibitors were not employed as controls; therefore, the causal relationship between NF-κB pathway suppression and the observed anti-inflammatory effects of 3PO remains to be confirmed.

## 5 Conclusion

Our data provide evidence supporting PFKFB3 as a viable target for asthma therapy. The anti-inflammatory effects of 3PO are linked to the suppression of NF-κB activation, which may contribute to the interruption of the cytokine cascade and cellular infiltration that drive asthma pathology.

## Data Availability

The datasets presented in this study can be found in online repositories. The names of the repository/repositories and accession number(s) can be found below: https://pubchem.ncbi.nlm.nih.gov/, 5720233 http://www.wwpdb.org/, 5R8Q http://www.wwpdb.org/, 7NXZ http://www.wwpdb.org/, 1TNF http://www.wwpdb.org/, 3GUT http://www.wwpdb.org/, 5UUK http://www.wwpdb.org/, 5IBP http://www.wwpdb.org/, 6PXR http://www.wwpdb.org/, 2XCG.

## Glossary

HK: Hexokinase
PFK-1: Phosphofructokinase-1
PK: Pyruvate kinase
F2,6BP: Fructose 2,6-bisphosphate
PFKFB3: 6-phosphofructo-2-kinase/fructose-2,6-bisphosphatase-3
IKB: Inhibitor of nuclear factor kappa B
IKK: IKB kinases
NF-κB: Nuclear factor kappa B
ELISA: Enzyme-linked immunosorbent assay
TNF-α: Tumor necrosis factor-α
IgE: Immunoglobulin E
CCL11: Chemokine (C-C motif) ligand 11
CCR3: Chemokine (C-C motif) receptor 3
IL-1β: Interleukin-1β
IL-4: Interleukin-4
IL-5: Interleukin-5
IL-13: Interleukin-13
IL-6: Interleukin-6
IL-17A: Interleukin-17A
IL-10: Interleukin-10
CTD: Comparative toxicogenomics database
KEGG: Kyoto encyclopedia of genes and genomes
GO: Gene ontology
BH: Benjamini–Hochberg
FDR: False discovery rate
SPF: Specific-pathogen-free
H&E: Hematoxylin& eosin
IF: Immunofluorescence
HRP: Horseradish peroxidase
DAB: Diaminobenzidine
BALF: Bronchoalveolar lavage fluid
PPI: Protein-protein interaction
ANOVA: One-way analysis of variance
3PO: 3-(3-pyridinyl)-1-(4-pyridinyl)-2-propen-1-one
BP: Biological process
CC: Cellular component
MF: Molecular function
CGenFF: CHARMM General Force Field
RMSD: Root mean square deviation
RMSF: Root mean square fluctuation
Rg: Radius of gyration
SASA: Solvent accessible surface area
